# Integration of Sustainable Carbon Nanoparticles Into Inverted Hybrid Perovskite Solar Cells to Enhance Optoelectronic Performance

**DOI:** 10.1002/gch2.202500363

**Published:** 2025-11-21

**Authors:** Lorenzo Squillantini, Davide Tocco, Marco Natali, Luca Gnoli, Alessio Mezzi, Chiara Dionigi, Eugenio Lunedei, Fabiola Liscio, Andrea Parisini, Mirko Seri, Samet Ocak, Silvia Milita, Emiliano Fratini, Giampiero Ruani, Francesca De Giorgio

**Affiliations:** ^1^ Consiglio Nazionale delle Ricerche Istituto per lo Studio dei Materiali Nanostrutturati (CNR‐ISMN) Bologna Italy; ^2^ Department of Chemistry ‘‘Ugo Schiff” & CSGI University of Florence Sesto Fiorentino (FI) Italy; ^3^ Consiglio Nazionale delle Ricerche Istituto per lo Studio dei Materiali Nanostrutturati (CNR‐ISMN) Montelibretti (RM) Italy; ^4^ National Reference Center for Electrochemical Energy Storage (GISEL) INSTM Via G. Giusti 9 Firenze 50121 Italy

**Keywords:** carbon nanoparticles, CNPs, environmentally friendly additives, hybrid perovskite solar cells, HPSCs

## Abstract

Hybrid Perovskite Solar Cells (HPSCs) using lead halide perovskites offer high performance and low‐cost fabrication via solution processes. However, their environmental and thermal instability, along with poor polycrystalline quality—such as trap states and grain boundaries—limit device efficiency. In this study, we propose four novel compositions of carbon nanoparticles (CNPs) as additives for methylammonium PbI_3_ (MAPI)‐based HPSCs to enhance the optoelectronic performance. The CNPs are synthesized through a green, cost‐effective method using citric acid and L‐tryptophan for nitrogen doping. Their optical, structural, and morphological properties are thoroughly characterized prior to integration. To assess the impact of CNPs on perovskite crystallization and facet orientation, synchrotron‐based 2D Grazing‐Incidence Wide‐Angle X‐ray Scattering (GIWAXS) is employed. Devices are fabricated using an inverted architecture, suitable for flexible substrates and energy‐efficient processing. Electrical and electrochemical impedance spectroscopy analyses reveal improved fill factors across all CNP compositions. The optimized system achieves a power conversion efficiency (PCE) of 10%, compared to 8.2% for the reference device without CNPs, confirming the potential of green CNPs to enhance HPSC performance without compromising structural integrity.

## Graphical abstract

Carbon nanoparticles (CNPs) synthesized from sustainable chemicals, that is, citric acid and L‐tryptophan, using a green synthesis method, are used as additives in methylammonium PbI_3_ (MAPI)‐based hybrid perovskite (HPSCs) solar cells. The use of CNPs improves the device's fill factor, giving power conversion efficiencies up to 10% in comparison to 8.2% of the benchmark system without CNPs.

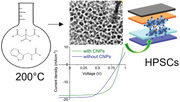

## 1 Introduction

Over the last decades, the so‐called third‐generation thin film‐based photovoltaic cells have continuously gained increasing attention [1, 2]. Within this class of devices, lead halide perovskite absorbers have been extensively studied due to their unique and specific properties compared to traditional silicon‐based materials, such as compatibility with flexible substrates, low‐cost solution processing, tunable bandgap, and high absorption coefficient [3].

Lead halide perovskite‐based solar cells have recently emerged, driven by a remarkable increase in their performance, reaching levels comparable to traditional inorganic‐based technologies [4, 5, 6].

However, these materials face significant challenges that limit their full potential, including environmental instability, lead toxicity, and difficulty in controlling the quality of the polycrystalline film. Grain boundaries and associated defects create charge traps and recombination sites, severely impacting the overall device performance. To address these issues, significant research efforts have been focused on developing strategies that leverage the solution processability of perovskite materials, such as solvent [7, 8, 9], additives [10, 11, 12, 13, 14, 15], and interfaces [16] engineering or passivation with lower dimensionality perovskites [17]. Among them, carbon‐based nanomaterials [18], including fullerene derivatives [19], carbon nanotubes [20], and graphene [21], were extensively investigated due to their excellent optical and charge transport properties. Carbon Quantum Dots (CQDs) are also gaining interest due to their simple synthesis procedures, which utilize low‐cost, abundant, and renewable precursors [22, 23, 24, 25].

In this context, it is evident that carbon nanoparticles (CNPs) are effective additives for lead halide perovskite absorbers when incorporated into the photoactive layer of perovskite solar cells. The chemical groups on their surfaces facilitate strong interactions with the grain boundaries of perovskite polycrystalline films, resulting in an improved power conversion efficiency (PCE) and long‐term stability of the corresponding devices. These enhancements are attributed to better crystallinity, larger grain sizes, improved surface quality, and defect passivation of the perovskite film, which contribute to higher fill factor (FF) values [26, 27, 28, 29, 30]. Furthermore, the chemical groups on the CNPs surface can be tuned by varying the reagents, which leads to different interactions between lead halide perovskite and CNP‐based additives, thereby modifying their charge transport properties. By employing two synthetic strategies to introduce distinct functional groups onto the film surface, Xu et al. demonstrated that the incorporation of alkyl groups significantly enhances the hydrophobicity of the CNPs, thereby improving both the performance and the stability of the resulting hybrid perovskite solar cells (HPSCs) [31].

Here, sustainable CNPs based on citric acid (CA), as a carbon source, were synthesized and integrated into the HPSCs active layer. Since nitrogen doping was demonstrated to improve the transport properties of CNPs [32], increasing amounts of a selected amino acid, like L‐tryptophan (Trp), were introduced into the CNP as a nitrogen source.

We present — for the first time to the best of our knowledge — a step‐by‐step investigation into the use of green CNPs as performance‐enhancing additives for MAPI‐based HPSCs to systematically evaluate the impact of CNPs at each level of integration.

First, we investigated four different CA:Trp weight ratios, that is, 100:0, 80:20, 75:25, and 70:30, hereinafter referred to as 100, 80, 75, and 70CA, respectively, for simplicity. A green synthesis method was employed to prepare CNPs under mild conditions, avoiding toxic chemicals and minimizing high temperatures. No purification steps were used to keep the process as simple as possible. A comprehensive and advanced characterization of the synthesized CNPs was conducted to analyze their optical, morphological, and structural properties.

Second, the CNPs were incorporated into the MAPI perovskite active layer, which is a prominent material for solar cells due to its excellent optoelectronic properties [33, 34], and it is widely studied, with extensive data and diverse characterizations available as a comparison, making it a well‐established benchmark for HPSC active layers. Therefore, although lead‐free perovskites have recently gained attention in the literature [35, 36, 37], we selected the MAPI‐based photoactive layer for this study to investigate how varying concentrations and chemical compositions of sustainable CNPs additives influence final device performance. To assess how the additives affect film formation and structural uniformity, we studied the role of CNPs in influencing the crystallization behavior and facet orientation under varying exposure times using synchrotron‐based 2D Grazing‐Incidence Wide‐Angle X‐ray Scattering (GIWAXS).

Finally, the optimized perovskite layers containing CNPs were integrated into HPSC devices fabricated using an inverted architecture. HPSC were fabricated on glass/indium‐tin‐oxide (ITO) substrate, using MAPI as the light‐absorbing material, poly(3,4‐ethylenedioxythiophene):polystyrene sulfonate (PEDOT:PSS) as the hole transport material (HTM), and phenyl‐C61‐butyric acid methyl ester (PC61BM) as the electron transport material (ETM), and tested under illumination. A thin layer of bathophenanthroline (BPHEN) was used as a buffer layer between ETM and back‐electrodes. This architecture is also suitable for the fabrication of flexible devices with reduced energy consumption, thanks to the lower annealing temperature required, that is, approximately 100 °C vs. 450 °C for inorganic ETMs like TiO_2_, as we previously demonstrated in Refs. [38, 39].

Electrical and electrochemical impedance spectroscopy analyses revealed an enhancement in the fill factor across all CNP compositions, with PCEs reaching up to 10%, compared to 8.2% for the reference devices without CNPs.

This step‐by‐step approach provides a comprehensive understanding of the role of green CNPs at each stage of device development, from additive material synthesis to full device performance.

## 2 Results and Discussion

### 2.1 Synthesis and Characterization of Carbon Nanoparticles

**FIGURE 1:**
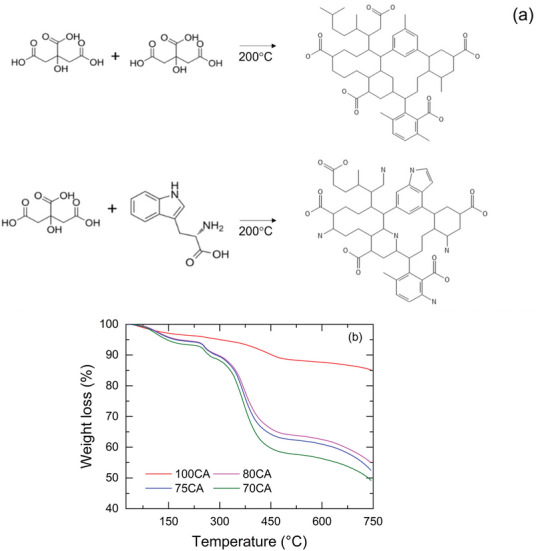
(a) Scheme of the CNPs’ synthesis; (b) TGA of the CNPs from RT up to 750 °C under N_2_ flux at the scan rate of 10 °C/min.

CNPs were synthesized via an easy‐scalable, cost‐effective, and sustainable process using the thermal cracking of citric acid (CA) in air and under atmospheric pressure [40]. Increasing amounts of L‐tryptophan (Trp) were added to the reaction mixture, leading to four different compositions (percentages refer to CA:Trp w/w%), namely 100:0 (100CA), 80:20 (80CA), 75:25 (75CA), 70:30 (70CA). The synthesis of the CNPs is illustrated in Figure 1a, where the CNPs molecular structures are only a schematic illustration and are not intended as a rigorous representation. The reaction was conducted above the CA melting point without using any solvent. Processes of carbonization, dehydration, and decarboxylation of the CA were the driving forces for the formation of the CNPs [40].

The Trp was added to provide additional functional groups and nitrogen doping to the CNPs. The as‐prepared CNPs were treated with aqueous NaOH to solubilize the CNPs, and HCl was used to neutralize the solution, resulting in NaCl as a by‐product. Since dialysis processes were not performed to maintain a sustainable, cost‐effective, and simple synthesis procedure, a fraction of NaCl remained in all the CNPs samples.

Thermogravimetric profiles of CNPs are shown in Figure 1b. Both the 100CA sample and the tryptophan‐modified CNPs exhibited a mass loss in the temperature range of 30 °C to 250 °C, due to water desorption and the formation of pyrolysis gases (e.g., CO_2_, CO, etc.) from functional groups likely located on the CNP surface [41]. The 100 CA sample showed a second mass loss step from 250 °C to 550 °C ascribable to the decomposition of the carbonaceous material [42]. In contrast, the 80, 75, and 70CA CNPs exhibited a mass loss in the range of 240 °C to 320 °C due to the thermal decomposition of tryptophan‐derived species [31]. In the range 240 °C–340 °C, samples 80, 75, and 70CA exhibit a mass loss of 5.8%, 6.0%, and 6.3%, respectively. This trend aligns well with the N/COOH ratios estimated by XPS measurements (Table 1). The final decomposition step occurred over the range of 320 °C – 550°C, likely associated with the degradation of the carbon core, including the elimination of phenyl groups [43].

**TABLE 1:** Nitrogen/carboxylic carbon atoms ratio extracted from C 1s and N 1s XPS spectra analysis.

CNPs	N/COOH by XPS analysis	N/COOH theoretical
100CA	–	–
80CA	0.23	0.15
75CA	0.29	0.20
70CA	0.36	0.25

**FIGURE 2:**
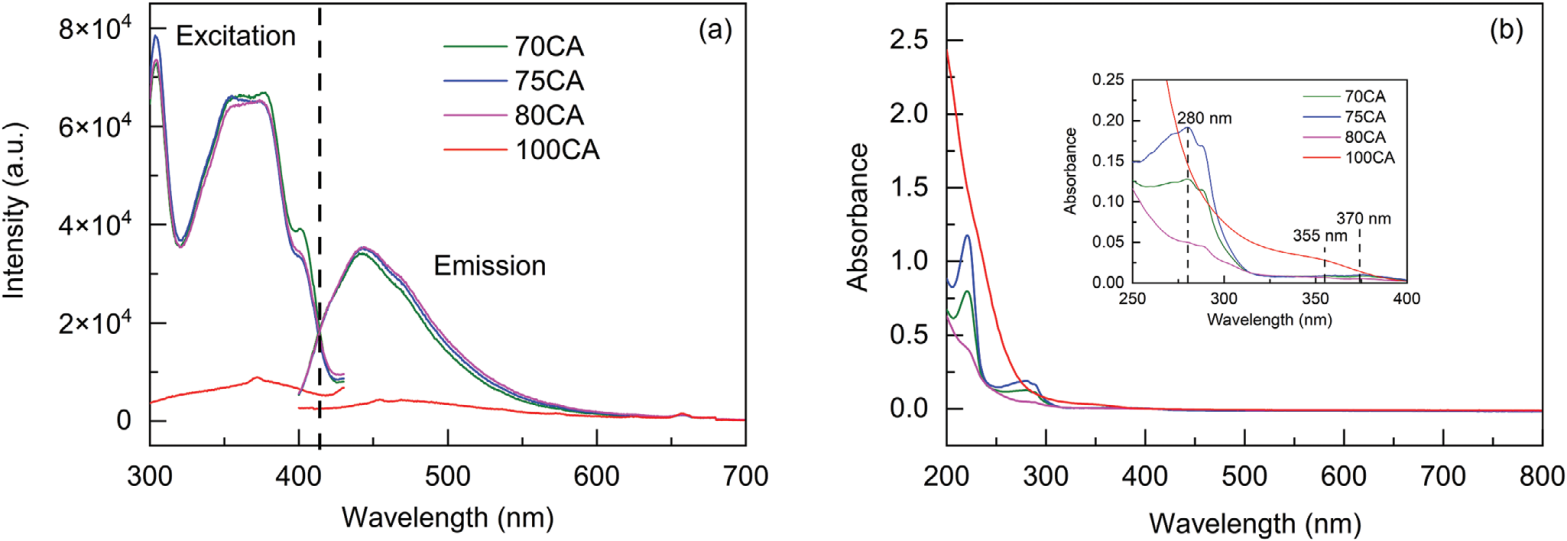
(a) Excitation (λ_em_ = 450 nm) and emission (λ_exc_ = 370 nm) spectra of CNPs; (b) UV–Vis absorption spectra of the CNPs. All spectra were recorded in water solutions.

The optical properties of CNPs dispersed in aqueous solution were investigated in terms of fluorescence and UV–Vis absorption. The solution containing the synthesized CNPs appears pale yellow in daylight, while it exhibits bright blue fluorescence under a 365 nm UV lamp. Upon excitation at 370 nm the fluorescence spectrum of the CNPs, shown in Figure 2a, reveals a strong and defined peak at 450 nm. This result agrees with data reported in the literature [40]. Notably, the 100CA composition shows a much weaker emission intensity, indicating that the nitrogen doping deeply affects the CNPs properties. Indeed, the indole group of Trp enables electron transfer to the surface and modifies the CNPs, enhancing the fluorescence [40].

Trp‐based CNPs exhibited an absorption band at 280 nm ascribable to the π−π* transitions of the indole ring characteristic of “isolated” Trp [40, 44], whilst the band at 370 nm stems from the n‐π* transition of the C═O [44]. The peak at 237 nm is due to a π−π*transition of the aromatic C═C bond [45]. In the 100CA sample, the n‐π* transition of the C═O is blue‐shifted to 355 nm compared to Trp‐CQDs (Figure 2b) [45].

**FIGURE 3:**
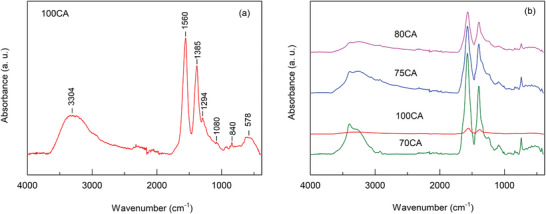
(a) ATR‐FTIR spectra of 100 CA and of (b) 70, 75, and 80CA compared to the spectrum of 100CA.

The ATR‐FTIR spectrum of the 100CA (Figure 3a) showed a broad absorption band centered at 3304 cm^−1^, attributed to the O─H stretching. Peaks at 1560, 1385, and 1294 cm^−1^ correspond to the asymmetrical and symmetrical stretching vibrations of the carboxylate ions (─COO^−^) and to the C─O stretching, respectively [46, 47]. The peaks in the 1294 – 1080 cm^−1^ region and at 840 cm^−1^ arised from the in‐plane and out‐of‐plane (“oop”) bending of the ring C─H bond in polycyclic aromatic hydrocarbon systems [47].

The successful incorporation of the Trp in the 80, 75, and 70CA compositions is proved through the appearance of additional features at ca. 1240 and ca. 740 cm^−1^ displayed in all the spectra (Figure 3b), corresponding to the aromatic C─N stretching and to the NH_2_ wagging and twisting, respectively [48]. The peak at approximately 1573 cm^−1^ is attributed to the antisymmetric bending modes of the H_2_N‐H, while the peak at 423 cm^−1^ refers to O–H stretching vibrations and N─H stretching vibrations associated with the amine groups [40].

**FIGURE 4:**
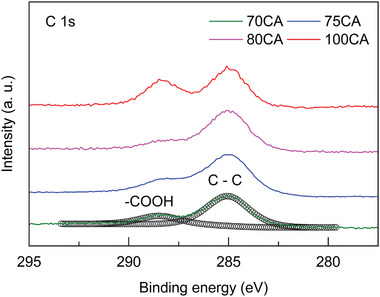
High‐resolution spectra of C 1s signals for all the synthesized CNPs.

The surface chemical composition of the samples was investigated using XPS to gain a deeper understanding of the chemical nature of the CNPs. Wide survey spectra of the samples (Figure S1a) confirmed the presence of carbon, chlorine, and sodium due to NaCl by‐product, as well as nitrogen (detected in all the samples except 100CA) and oxygen. High‐resolution spectra were recorded for all detected elements to examine their chemical environments. For all CNP compositions, the C 1s peak was fitted with two components (Figure 4) positioned at binding energy (BE) 285.0 and 288.5 eV, corresponding to C‐C bond and to carboxylic COOH functional groups, respectively.

The signals of O 1s (Figure S1c) and N 1s (Figure S1b), which are present in all the CNPs except 100CA, were fitted with a single component, assigned to C═O (531.5 eV) and amine (399.9 eV), respectively. The ratio of nitrogen stoichiometric content to the carbon component assigned to ─COOH functional group (Table 1) was calculated and compared to the expected theoretical values to assess nitrogen incorporation in the CNPs. Experimental values were higher than the theoretical ones in all cases, likely due to decarboxylation, carbonization, and dehydration processes during CNP synthesis, resulting in a partial loss of COOH groups. Nevertheless, an increase in nitrogen content with higher Trp amounts in the reaction mixture confirmed the successful incorporation of nitrogen into the CNPs. All atomic percentages and BE are provided in Table S1.

**FIGURE 5:**
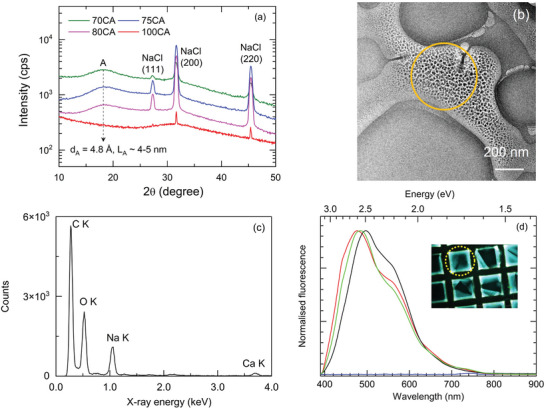
(a) XRD patterns of the synthesized CNPs; (b) bright‐field (BF) TEM micrograph of dialyzed 70CA sample, (c) EDS spectrum obtained on the region marked with a circle in (b); (d) emission spectra of 70CA samples (λ_exc_ = 365 nm) as recorded from dialyzed CNPs deposited on TEM grid (red line; inset, 70 µm‐diameter‐wide signal collection area, yellow circle) and on glass (green line). The emission spectrum of non‐dialyzed CNPs deposited on glass (black line) is traced for comparison. The signal as collected from an empty grid is shown (blue line).

The absence of significant fractions of graphitic sp^2^ carbon atoms was further confirmed by XRD patterns, which did not display the characteristic graphite peak at 20° (Figure 5a). However, all samples exhibited a broad peak at around 18° with a d‐spacing of 4.8 Å and coherence lengths of 4–5 nm [49, 50, 51], calculated using the Debye‐Scherrer equation [52]. This feature can be attributed to the presence of CNPs, and the absence of a clear crystalline structure is presumably due to the relatively mild reaction conditions, that is, low temperatures and atmospheric pressure. Additionally, the XRD patterns of all the samples revealed characteristic peaks associated with NaCl crystal structures.

To deeply investigate CNP structure and dimensions, TEM analysis was performed on 70CA since it was the best‐performing additive in HPSCs’ active layer.

High‐resolution electron microscopy (HREM) investigations clearly revealed the presence of crystalline nanoparticles, characterized by lattice fringes spaced at 0.33 and 0.28 nm (Figure S2b,d). In Figure S2a,c, the diffractograms obtained from these HREM micrographs display diffraction spot patterns that can be confidently indexed to the main Halite (NaCl) reflections (rightmost panel of Figure S2).

Furthermore, X‐ray energy‐dispersive spectroscopy (EDS) performed on the same regions observed by HREM confirmed the presence of chlorine and sodium, as shown in the EDS spectrum of Figure S3a. A comparison between experimental and simulated (S3b) EDS spectra revealed an excess of sodium in the sample, beyond what would be expected from the NaCl particles alone.

In summary, the broad peak around 18° observed in XRD, along with the apparent absence of other crystalline features aside from the NaCl particles seen in TEM, suggests that the CNPs, a few nanometers in size, are present in an amorphous or weakly crystalline structure that may incorporate the excess sodium detected by EDS [53].

To investigate the CNPs in the absence of NaCl particles, we characterized samples with the same CA:Trp ratio after dialysis treatment. In this case, no crystalline structures were detected by electron diffraction. Instead, as shown in the bright‐field micrograph of Figure 5b, amorphous nanoparticles ranging from a few to several tens of nanometers were observed. As illustrated in the BF micrographs of Figure S4, these particles exhibited a clear tendency to grow and agglomerate under electron irradiation during TEM observations. This behavior likely resulted from increased atomic mobility induced by the electron beam–sample interaction, suggesting that particle formation may occur in situ from smaller nuclei.

Figure 5c shows an EDS spectrum acquired using a relatively large electron beam, with the position and diameter of the analyzed area indicated in Figure 5b. This analysis revealed the presence of C, O, Na, and a trace of Ca as contaminants, with no detectable Cl. The persistence of sodium after dialysis is likely due to the salification of the –COOH functional groups on the CNPs. The loss of these functional groups through electron beam sputtering may explain the observed growth and coalescence of the CNPs under conditions of high atomic mobility (Figure S4).

Figure 5d shows the microfluorescence spectra (λ_exc_ = 365 nm) of dialyzed 70CA sample deposited by drop casting from a methanol suspension (volume: 5 µL) on a TEM grid (red line) and on glass (green line). The fluorescence signal was selectively collected, under the microscope by using a 50x objective, from a circular area (diameter 70 µm, yellow dashed circle of Figure 5d inset) which fits the TEM grid square aperture dimension. For the sake of comparison, the fluorescence spectrum of a non‐dialyzed 70 CA sample on glass is also shown (black line). The spectra of the dialyzed samples are nearly independent of the substrate, but they exhibited a blue shift of ca. 0.1 eV with respect to the spectrum of the non‐dialyzed sample, probably due to a different chemical environment after dialysis processes.

### 2.2 Characterization of Perovskite Films and HPSCs

Prior to evaluating the performance of MAPI‐based perovskites in HPSCs, we conducted Grazing Incidence Wide Angle X‐ray Scattering (GIWAXS) measurements on MAPI‐based films to assess their structural stability over time when exposed to air. 2D‐GIWAXS images of MAPI‐based films, with and without the optimized 70 CA composition (0.25 mg/mL) deposited on PEDOT:PSS layers, were collected after various exposure times, that is, 0, 24, and 48 h, under ambient conditions (room temperature (RT) and relative humidity (RH) of 50%).

**FIGURE 6:**
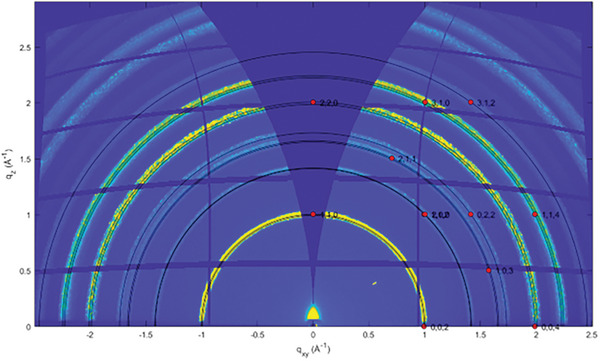
2D‐GIWAXS image of the MAPI‐based film on PEDOT, recorded using an incident angle of 0.3° and an X‐ray wavelength of 1.4 Å. The hkl reflections are indexed assuming the orthorhombic crystal phase.

For both film types, all recorded diffraction signals could be indexed with the orthorhombic crystal phase of MAPI (a = 8.849 Å, b = 8.849 Å, c = 12.642 Å; a = b = g = 90°), which is known to be stable at RT. This confirmed the absence of contaminants or residual precursors (Figure 6; Figure S5) and indicated that the incorporation of CNPs does not alter the crystal structure of the film. Moreover, the nearly uniform distribution of scattered intensity along the Debye rings indicated that the MAPI crystallites are randomly oriented with respect to the substrate.

To probe the depth‐dependent structural homogeneity, a series of images was acquired using varying incident angles of the X‐ray beam (from 0.05° to 0.3°), corresponding to penetration depths ranging from 5 nm to 1 µm. The invariance of the observed structural features across these depths confirmed the uniformity of the films throughout their thickness, both for the film without (Figure S6a) and with 70CA (Figure S6b).

The 2D‐GIWAXS images recorded after air exposure of MAPI films (neat Figure S7a and with 70 CA Figure S7b) for 24 and 48 h clearly showed that their crystalline structure remains unchanged, with no signals indicative of degradation‐related compounds or precursors, although partial amorphization or desorption must be inferred from the slight decrease in diffracted intensity.

The comparable structural stability observed in both systems suggested that the differing performances of the final HPSCs—10% PCE with 70CA and 8.2% PCE without CNPs—cannot be attributed to variations in structural evolution, which in both cases corresponds to that of MAPI itself. This finding confirmed that the presence of CNPs does not affect the crystalline structure of the perovskite over time.

**FIGURE 7:**
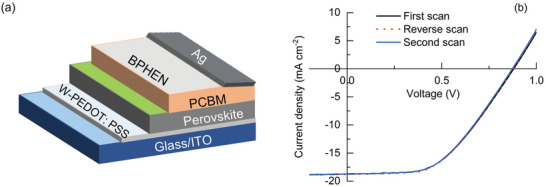
(a) Scheme of the PSCs architecture; (b) *J/V* curves of the pristine champion device.

MAPI‐based HPSCs were fabricated by using a one‐step, anti‐solvent assisted process [54, 55, 56]. The device architecture consisted of a PEDOT:PSS layer as HTM, a PC61BM layer as ETM, and a thin layer of BPHEN as interfacial improver between ETM and back‐electrodes (Figure 7a). The washing step of the W‐PEDOT:PSS layer removes a fraction of PSS from the surface, thereby improving the transport properties of the HTM and reducing its acidity [57]. This treatment has also been demonstrated to improve the device's stability in air and over time [58].

A set of six pristine devices was fabricated and tested to establish the performance of the benchmark MAPI‐based HPSCs in terms of PCE and electrical properties. The average PCE of the tested devices was 8.1% ± 0.1, with the champion device achieving a PCE of 8.2% PCE, FF of 50%, open‐circuit voltage (*V_OC_*) of 0.88 V, and a short‐circuit current density (*J_SC_*) of 18.7 mA/cm^2^, consistent with the values reported in the literature for similar device architectures [59, 60, 61]. Negligible hysteresis effects were observed by performing reverse scans (Figure 7b).

**FIGURE 8:**
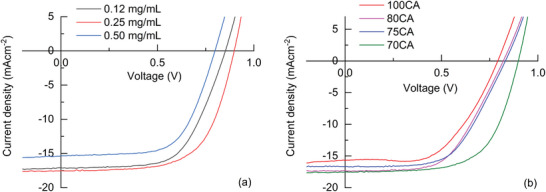
Comparison of the *J* vs. *V* curves obtained in reverse scan (from 1.0 to −0.5 V at 100 mV/s scan rate) under solar irradiation of the champion HPSCs incorporating (a) 70CA at different concentrations (0.12, 0.25, and 0.50 mg/mL) and (b) 100, 80, 75, and 70CA at the fixed concentration of 0.25 mg/mL.

The effect of CNPs as additives on HPSCs was first investigated starting from 70 CA, since it features the highest nitrogen doping that was described as beneficial for charge transport properties of carbon nanoparticles [61]. The chosen concentrations were 0.12, 0.25, and 0.50 mg/mL, accordingly to similar reported values for CNPs used as additives in HPSCs (Figure 8a) [27, 29, 31, 62]. The best performance was achieved with a concentration of 0.25 mg/mL, where the champion device exhibited a PCE of 10%, along with improvements in FF (63%) and *V_OC_* (0.90 V). Average values, calculated on six different devices, exhibited a significant improvement compared to the pristine one (Table 2). Indeed, PCE passed from 8.1% ± 0.1 to 9.2% ± 0.4, and the FF from 53.5% ± 1.5 to 60% ± ss3. At the lowest concentration (0.12 mg/mL), only a moderate improvement in overall HPSC performance was observed for the champion device compared to the pristine (PCE 9.0% vs. 8.2%), but on average, the values are lower (Table S2). Conversely, at the highest concentration (0.50 mg/mL), a slight decrease in PCE was recorded for the champion device (7.8%), with an average value lower than the pristine (7.1 ± 0.3% vs 8.1 ± 0.1%) but a significant increase in the average FF (61% ± 3 vs 53.5% ± 1.5) (Table S2), even slightly better than the optimized concentration (0.25 mg/mL). These data indicated that a low amount of CNPs is not sufficient to significantly improve HPSCs performances, while an excess of CNPs could lead to adverse effects on transport properties, leading to lower *V_OC_* and *J_SC_* but not on the FF. This could be due to a beneficial effect of CNPs on the passivation of the perovskite film and on the quality of the interfaces composing the device.

**TABLE 2:** Average *J_SC_*, *V_OC_*, FF, and PCE figure of merit calculated from six different HPSCs devices with CNPs at different compositions (100, 80, 75, 70CA, and 0.25 mg/mL concentration) obtained under solar irradiation in reverse scan. In brackets, the values of each champion device are reported (Figures 6 and 7b).

Additive in HPSC	*J_SC_* (mA cm^−2^)	*V_OC_* (V)	FF (%)	PCE (%)
No additives	17.7 ± 0.4 (18.7)	0.85 ± 0.01 (0.88)	53.5 ± 1.5 (50)	8.1 ± 0.1 (8.2)
70CA	17.8 ± 0.6 (17.5)	0.86 ± 0.03 (0.90)	60.0 ± 3.0 (63)	9.2 ± 0.4 (10.0)
75CA	16.3 ± 0.4 (16.7)	0.82 ± 0.01 (0.83)	53 ± 2.8 (58)	7.1 ± 0.6 (8.0)
80CA	16.1 ± 0.8 (17.3)	0.84 ± 0.01 (0.82)	55.1 ± 1.4 (56)	7.5 ± 0.4 (8.0)
100CA	14.5 ± 1 (15.7)	0.77 ± 0.01 (0.79)	52.4 ± 2.1 (56)	5.9 ± 0.7 (7.0)

Figure S8 shows the external quantum efficiency (EQE) spectra of the best‐performing devices incorporating CNPs at concentrations of 0.25 and 0.50 mg/mL. The corresponding theoretical *J_SC_* values, which were calculated from the convolution of the EQE profiles with the AM1.5G solar spectrum, are consistent with those obtained experimentally from the *J‐V* measurements. The onset of photocurrent at 800 nm aligned with the optical bandgap of MAPI perovskite (E_g_ = 1.55 eV), demonstrating the successful absorption and utilization of photons across the spectrum.

To further investigate how the performance of HPSCs is influenced by varying the nitrogen content of the CNPs, the fixed concentration of 0.25 mg/mL — previously identified as optimal — was applied to all CNP compositions (100, 80, 75, and 70CA). The resulting PCEs calculated from *J* vs. *V* curves in forward and reverse scans (Figure S9) were similar for the 80 and 75CA compositions, while a lower value was calculated for 100 CA. For the 70CA composition, the higher *V_OC_* positively influenced the PCE, resulting in the highest performance among the tested HPSCs (Figure 8b and Table 2). These findings suggest that the N‐doping helps to passivate grain surface trap states, thereby contributing to the enhanced *V_OC_* of the device [63, 64, 65].

An increase in the FF was observed in the champion devices for all compositions, rising from 50% for the pristine device to approximately 60% for devices containing CNP additives, while average values are similar to the pristine devices. However, *J_SC_* values of the HPSCs featuring CNPs at different compositions and 0.25 mg/mL concentration were lower compared to the pristine device for the champion devices but slightly higher for the average value of the 70CA (Table 2). This effect was likely due to the influence of CNPs on the morphology of the perovskite polycrystalline film and their transport properties. In particular, the chemical groups present onto the CNP surfaces, including carboxylic and nitrogen‐based groups, may contribute to an increase in the average crystalline grain size. This, in turn, can lead to improved shunt and series resistance values, ultimately enhancing FF. Additionally, nitrogen doping may further enhance the electron transport properties of CNPs [28, 32, 58, 66], potentially increasing the current density compared to other CNP compositions, though not surpassing that of pristine perovskite.

All the results reported in Table S2 and Table S3 are summarized in Figure S10.

For clarification, to evaluate the effect of NaCl by‐product present in the CNP additive samples, 0.06 mg/mL of NaCl was added to the perovskite solution to match the same salt content of the optimal 0.25 mg/mL‐70CA formulation. The salt content in CNP samples was estimated to be approximately 25% by total weight. Devices with and without NaCl exhibited comparable PCEs (Figure S11), demonstrating that the by‐product did not affect the performance of HPSCs.

**FIGURE 9:**
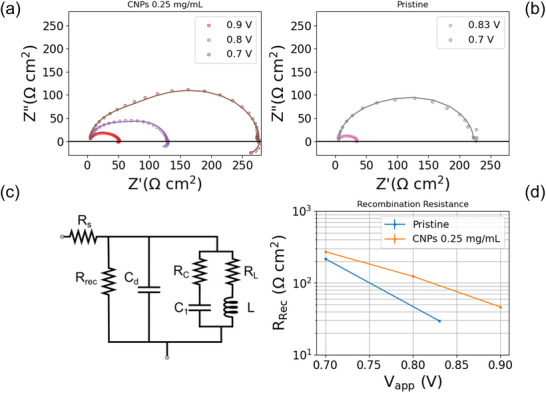
Nyquist plots of HPSCs (a) featuring CNP additives (70CA) at the optimized concentration of 0.25 mg/mL and of (b) the pristine one; (d) equivalent circuit model used for the EIS data fitting; (e) R_rec_ with respect to the applied voltage.

To gain further insight into the electronic behaviour of the HPSC devices, we conducted electrochemical impedance spectroscopy (EIS) analysis under dark conditions at various applied voltages (V_app_) up to *V_OC_*. Figure 9 displays the EIS spectra of the HPSC with the optimal 0.25 mg/mL‐70CA formulation (Figure 9a), which showed the highest PCE, as well as the spectra of the pristine device (Figure 9b) for comparison.

Figure 9c shows the equivalent circuit (EC) used to fit the EIS experimental data [67], where R_s_ is the series resistance, C_d_ is the dielectric capacitance, R_rec_ is the recombination resistance, R_L_ corresponds to a resistance associated with the inductance L, and R_C_ is the series resistance associated with the interfacial charging capacitance C_1_.

This model was successfully employed to describe the electrochemical performance and complex ionic behavior of HPSCs based on MAPbI_3_ perovskites [68].

The EIS analysis evinced the different recombination resistances between the two devices (Figure 9d; Table S3).

R_rec_ is a charge transfer resistance that can be measured even under dark conditions by applying a DC voltage, following the superposition principle approximation [69].

Notably, the device featuring CNPs exhibited higher R_rec_ values (Table S3), indicating a reduction in recombination losses due to the presence of CNPs. This reduction demonstrates the superior optoelectronic performance of the CNP‐based HPSC compared to the pristine one. Additionally, the C_d_ parameter, related to the device's dielectric properties, was higher for the CNP‐based device, suggesting an improved structural quality of the perovskite CNPs, as previously reported in literature [66].

Negative capacitance and inductive loops were observed in all the EIS spectra at low frequencies, represented as an inductance in the R_L_−L line of the equivalent circuit (Figure 9c). This phenomenon may be attributed to the interaction between ionic and electronic effects, leading to delayed dynamics, as well as to the device's intrinsic instability during the EIS measurement [67].

## 3 Conclusions

Carbon nanoparticles (CNPs) with four different compositions were synthesized using sustainable and cost‐effective precursors, that is, citric acid (CA) and L‐tryptophan (Trp), through a simple, scalable, and green process developed under mild and environmentally friendly conditions. The resulting CNPs were characterized and then incorporated as additives in MAPI‐based hybrid perovskite solar cells (HPSCs), leading to increased fill factor (FF) of the devices for all compositions with respect to the HPSCs without additives. Notably, CNPs composed of 30% Trp and 70% CA (w/w %) significantly improved the key electrical parameters, particularly the power conversion efficiency (PCE). This optimized composition enables HPSCs with increased PCE from 8.2% in the benchmark device to 10%, thanks to enhanced optoelectronic and charge transport properties, as confirmed by the electrochemical impedance spectroscopy (EIS) analysis. Furthermore, the 2D Grazing‐Incidence Wide‐Angle X‐ray Scattering (GIWAXS) experiments on 70CA‐MAPI‐based perovskite layers deposited on PEDOT:PSS confirmed that the incorporation of CNPs does not alter the crystal structure of the films, even under different exposure times in ambient conditions.

This study demonstrates that using low‐cost, sustainable CNP additives is a viable and promising strategy for enhancing the performance of MAPI‐based HPSCs. Additionally, this approach holds potential for application in lead‐free HPSC systems, offering a pathway to more environmentally friendly and efficient solar cells.

Further investigations are underway to understand how CNPs and their functional groups influence perovskite crystallization and facet orientation, to identify feasible routes for enhancing both the efficiency and stability of HPSCs.

## 4 Experimental Section

### 4.1 Materials

Lead iodide (PbI_2_, 99.99%) was purchased from Alfa‐Aesar. Methylammonium iodide (MAI) was purchased from Greatcell Solar Materials. Phenyl‐C61‐butyric acid methyl ester (PC61BM) was purchased from Solenne BV. Bathophenanthroline (BPHEN) was purchased from Lumtec. Poly(3,4‐ethylenedioxythiophene) doped with polystyrene sulfonate (PEDOT:PSS) was purchased from Heraeus (Clevios, P VP Al 4083). Silver (Ag, 99.99%) was purchased from Nanovision Srl. Substrates covered with indium‐tin‐oxide (ITO) were purchased from Visiontek Systems Ltd. Citric acid monohydrate (> 99.5 %, CAM, CAS: 5949‐29‐1) was purchased from MERK; L‐Tryptophan, (> 99.5 %, CAS: 73‐22‐3) was purchased from FLUKA. All the other materials and solvents, such as acetone, 2‐propanol, dimethylformamide (DMF), dimethyl sulfoxide (DMSO), chlorobenzene (CB), dichlorobenzene (DCB), sodium hydroxide (NaOH), and hydrochloric acid (HCl, 37%) were purchased from Sigma‐Aldrich and were used as received without further purification.

### 4.2 Carbon Nanoparticles Synthesis

The synthesis of CNPs was carried out following the procedure reported by Zhang et al., with further modifications [40]. The CNPs sample, labeled 100CA, was synthesized using citric acid as the only carbon source, without the addition of tryptophan. Afterward, a variety of samples were prepared by adding different amounts of tryptophan (0.25, 0.33, and 0.43 g per gram of citric acid) to investigate the effect of increasing nitrogen content. Briefly, CNPs were synthesized via a thermal cracking method. An aliquot of citric acid monohydrate (for 100CA) or a mixture of citric acid monohydrate and tryptophan (for the doped samples) was placed in a 40 mL vial and heated in an oil bath at 200 °C under continuous stirring (100 rpm). The reagents completely melted into a colorless liquid within 5 min. A small amount of water (2 mL, added 10 times) was introduced to prevent complete carbonization of the reaction mixture. The color of the mixture gradually changed from pale yellow to orange within 45 min, indicating the formation of CNPs (dense sticky liquid that later solidified at room temperature). The resulting mixture was dissolved in 10 mL of NaOH solution (1 M), and the pH was adjusted to 7.0. Finally, the product was freeze‐dried and stored at –18 °C. Sample 70CA (0.430 g of tryptophan per gram of citric acid) was further purified by dialysis using a 12–14 kDa molecular weight cut‐off (MWCO) dialysis membrane.

### 4.3 Device Fabrication

The HPSCs were fabricated in a clean room facility, starting from the cleaning of the glass/ITO substrates (size: 25 mm × 18 mm). The substrates were cleaned with deionized water, acetone, 2‐propanol for 5 min each. Subsequently, the substrates were treated with oxygen plasma (200 W, 5 min). Then, PEDOT:PSS was spin‐coated in air onto the ITO surface at 4000 rpm for 40 s (acceleration 4000 rpm/s). The PEDOT:PSS layer was further treated by blade coater washing with 2‐propanol onto the surface (hereinafter referred to as washed PEDOT:PSS, W‐PEDOT:PSS), followed by annealing for 20 min at 80°C. The MAPI active layer and all the other layers of the devices were deposited (and their solutions prepared) in a N_2_‐filled glovebox with oxygen and water levels below 10 ppm and 1 ppm, respectively. The MAPI solution was prepared at 1 M concentration for both PbI_2_ and MAI in a solvent mixture of DMF:DMSO (4:1). The PC61BM solution was prepared at a 20 mg/mL concentration in chlorobenzene: dichlorobenzene (3:1) solvent mixture, and BPHEN was dissolved in 2‐propanol with a 0.5 mg/mL concentration.

CNPs with various compositions and concentrations were first dissolved in the 4:1 DMF:DMSO solvent mixture for the CNP‐based devices. The resulting suspensions were sonicated for 30 min and then stirred overnight at 90°C in the glovebox. PbI_2_ and MAI were then added. All solvents were anhydrous and stored in the glovebox. The MAPI perovskite layer was formed by pouring 50 µL of the solution onto the glass/ITO/W‐PEDOT:PSS substrate. It was deposited using a spin‐coating process in two sequential steps: the first at 2000 rpm (acceleration 2000 rpm/s for 10 s) and the second at 5000 rpm (acceleration 3000 rpm/s for 20 s). During the final 2 s of the second step, 150 µL of chlorobenzene was dripped onto the film as an anti‐solvent. Thermal annealing of MAPI film was performed at 100°C for 5 min. The PC61BM layer was deposited by spin‐coating at 1000 rpm (acceleration 500 rpm/s for 20 s), followed by annealing at 100°C for 3 min. BPHEN was deposited at 4000 rpm (2000 rpm/s acceleration for 20 s), followed by annealing at 100°C for 1 min. Finally, the fabrication of the device was completed by thermally evaporating 100 nm of Ag for the electrical contact at a pressure of approximately 6 × 10^−6^ mbar.

### 4.4 Characterization of the Carbon Nanoparticles

UV/Vis, fluorescence, and emission spectroscopy: It was performed on freeze‐dried samples, re‐dissolved in Milli‐Q water (0.5 mg/mL in a cuvette). Absorption spectra were recorded using a UV/Vis Agilent Cary 300 spectrophotometer in the range of 200–800 nm. Fluorescence emission spectra were recorded using a spectrofluorometer FS5 v2 (Edimburgh Instruments). The spectra were acquired in the range of 360–690 nm upon excitation at 370 nm, at 25 °C. Each spectrum represents the average of three independent scans.

Emission spectra of CNPs as well as their fluorescence images were recorded using a modified Nikon Eclipse80i epifluorescence microscope. The samples were deposited on glass substrates or on properly prepared TEM grids. The standard trinocular turret of the microscope was customized to mount a home‐made optical system (composed of a 50 mm focal quartz lens focused into a UV–Vis/NiR optical fiber (200–2200 nm), 550 µm core, Thorlabs) to feed the fluorescence signal into an Avantes AvaSpec‐2048 CCD spectrometer (2048 pixels array, DLC UV/Vis, 200‐ 1100 nm range, 10 µm or 100 µm entrance slit, software programmed (further details can be found in Ref [70]). This allowed to record fluorescence images and collect the photoluminescence signal from CNPs under a 20x‐100x objective. In reflection imaging mode, the built‐in Nikon top halogen lamp was used (white light, illumination range 380–1200 nm). The lamp light reflected by the sample's surface was then collected through the microscopy objective and fed into the spectrometer fiber. For the photoluminescence spectra, a top‐mounted OSRAM Mercury Short Arc lamp (HBO) 100 W was properly energy filtered to provide a suitable excitation (λ_exc_ = 365 nm) to excite the CNPs across the microscope objective. The fluorescence signal collected back across the same objective was fed into the spectrometer through a dichroic mirror and a longpass filter to suppress residual excitation straylight. Within the same experimental session, it was possible to select the more suitable sampling area by visual inspection under strong magnification, then record a bright‐field microimage, followed by a fluorescence image and the local micro‐fluorescence spectrum. Fluorescence images (collected using a Nikon DigitalSight DS‐ 2 M camera) and emission spectra were recorded by using a 365 nm excitation filter, a 400 nm dichroic mirror, and a 420 nm long‐pass exit filter. For fluorescence spectra, integration time was of the order of 500 ms.

Thermogravimetric Analysis (TGA): Thermogravimetric Analyses (TGA) were performed using a Discovery SDT 650 (TA Instruments) in a temperature range of 25°C to 700°C with a heating rate of 10°C min^−1^ under continuous nitrogen flow (flow rate = 40 mL min^−1^).

Attenuated Total Reflectance‐Fourier Transform Infrared Spectroscopy (ATR‐FTIR): The measurements were conducted using a nitrogen purged Bruker Vertex 70 interferometer equipped with a DLaTGS detector, and a KBr beamsplitter. A single reflection diamond crystal‐based Platinum‐ATR accessory was employed. Spectra were recorded at RT in the 400 ‐ 4000 cm^−1^ spectral range with a resolution of 4 cm^−1^. Spectra were subsequently modified by applying extended ATR correction.

X‐ray photoelectron spectroscopy (XPS): The surface chemical compositions of the samples were investigated by an ESCALAB MkII (VG Scientific Ltd, East Grinstead, UK) equipped with a standard Al excitation source (hν = 1486.6 eV), a hemispherical analyser and 5‐channeltron detection system. The measurements were performed operating at constant pass energy CAE = 50 eV, and at a base pressure of 1 × 10^−10^ mbar. The samples were introduced in the apparatus, fixing them on a pure gold foil (99.9 wt%) by mechanical pressure with a stainless steel spatula. The binding energy (BE) scale was calibrated, positioning the C1s peak from adventitious carbon and the Au 4f_7/2_ peak at 285.0 and 84.0 eV, respectively. The XPS data were gathered and processed by the software Avantage v. 5.9 (Thermo Fisher Scientific Ltd).

Transmission electron microscope (TEM): The samples were prepared by depositing drops (about 5 µL) of methanol suspensions containing the synthesized CNPs onto ultrathin carbon films (with a nominal thickness of 3 nm) lying across lacey carbon films supported by Au TEM grids. An FEI Tecnai F20 microscope was used to investigate the morphology and the structure of the deposits by bright field and high‐resolution TEM (BF and HRTEM). For the observation of C‐rich nanomaterial on C support films, the electron microscope was operated at an accelerating voltage of 200 kV either to preserve signal‐to‐noise ratio or to limit the electron beam damage by inelastic scattering. A consequence of this choice is the unavoidable presence of atomic displacement damage owing to the elastic interactions of the incident electrons with the atomic nuclei of the target that we tried to face by limiting the electron dose.

X‐ray diffraction (XRD): The measurements were carried out using a Rigaku SmartLab diffractometer equipped with a rotating Cu anode, a CBO unit, and Soller slits to produce a parallel beam with a divergence of 0.1°. The synthesized CNPs were placed on glass powder sample holders for analysis.

### 4.5 Characterization of the Perovskite Films and HPSCs

Grazing Incidence X‐ray Diffraction (GIWAXS) measurements: To investigate the structural stability of the MAPI‐based perovskites over time when exposed to ambient air, 2D‐GIWAXS images of MAPI‐based films, with and without the optimized 70CA composition (0.25 mg/mL), deposited on PEDOT:PSS layers, were collected after various exposure times (0 h, 24 h, and 48 h) at RT and RH of 50%. The measurements were performed at the XRD1 beamline of the ELETTRA synchrotron radiation facility in Trieste (Italy), by using an X‐ray beam of 1,4 Å wavelength, with a size of 200 × 200 µm^2^ and a 2 M Pilatus silicon pixel X‐ray detector (DECTRIS Ltd.) positioned perpendicular to the incident beam, 200  mm away from the sample. The images were collected with incident angles of the X‐ray beam α_i_, from 0.05° to 0.3° to probe the uppermost film layers and the full film thickness. The visualization and analysis of the collected 2D‐GIWAXS images have been performed by means of the GID‐Vis software [71].

Optoelectronic characterization: To test the photovoltaic performance of HPSCs under solar irradiation, both pristine and CNPs‐based devices were measured using a Keithley 236 source‐meter unit to obtain current density–voltage *(J–V)* curves. Illumination was provided by a solar simulator (AM1.5G at 100 mW cm^−2^, Abet Technologies Sun 2000 Solar Simulator). During measurements, each HPSC was covered with a calibrated mask (0.0587 cm^2^) to exclude any additional photocurrent from parasitic regions outside the overlap of the Ag and ITO electrodes. The *J–V* curves were recorded with the devices maintained inside a glove box to ensure an oxygen‐ and moisture‐free environment. A voltage scan was performed at 100 mV/s scan rate, ranging from −0.5 to 1.0 V and in reverse mode from 1.0 to −0.5 V, to investigate hysteretic effects and eliminate transient effects due to ionic motion. The key performance parameters derived from the *J–V* curves include the short‐circuit current density (*J_SC_*), the open‐circuit voltage (*V_OC_*), the fill factor (FF), and the power conversion efficiency (PCE).

The external quantum efficiency (EQE) of the investigated HPSCs was measured in air between 300 and 900 nm (with a wavelength step size of 2 nm) using a custom‐built system controlled via LabView software. Monochromatic light was generated by a xenon arc lamp (Lot‐Oriel, 300 W) coupled with an automated monochromator (Spectra‐Pro). The light beam was modulated using an optical chopper operating at 80 Hz. The photocurrents generated in the HPSCs and in a calibrated silicon photodiode, used as a reference, were recorded using a digital lock‐in amplifier (Stanford Research Systems SR830). Theoretical *J_SC_* values were calculated by convolving the EQE profiles with the AM1.5G solar spectrum, showing good agreement with the experimentally measured *J_SC_* values from *J‐V* measurements under illumination.

Electrochemical characterization: The electrochemical impedance spectroscopy (EIS) measurements were performed on pristine MAPI‐ and CNPs‐based HPSCs under different DC voltages using an AUTOLAB potentiostat/galvanostat with an AC amplitude of 10 mV, and the data were collected by taking 10 measurements per frequency decade in the range of 1 MHz – 10 (or 20) mHz. The EIS measurements were carried out at RT in an N_2_‐filled glovebox under dark conditions to avoid any heating phenomena that can be generated under solar simulator irradiation. PyEIS software was used for EIS data fitting.

## Funding

Mission Innovation Program under the Grant “Italian Energy Materials Acceleration Platform—IEMAP” — Ministero dello Sviluppo Economico (MiSE); PNRR MUR project ECS_00000033_ECOSISTER, project funded under the National Recovery and Resilience Plan (NRRP), Mission 04 Component 2 Investment 1.5 – NextGenerationEU, Call for tender n. 3277 dated 30/12/2021. Award Number:0001052 dated 23/06/2022; Prin 2022 “DiGreen: A digital and chemical approach for green recycling of Li‐based batteries” (no. 2022W37L2L)—MUR (Ministero Italiano dell'Università e della Ricerca); Regione Toscana (FSC 2014‐2020, GiovaniSi acronym project QD4PH, CUP: B13D21008630008, FSE: 291452); Prin 2022 “LUminescent modular NAno‐composites for Responsive LIGHTing devices (LUNARLIGHT)” (no. 2022NHLX2M)—MUR (Ministero Italiano dell'Università e della Ricerca).

## Conflict of Interest

The authors declare no conflict of interest.

## Supporting information




**Supporting file**: gch270069‐sup‐0001‐Suppmat.pdf

## Data Availability

The data that support the findings of this study are available from the corresponding author upon reasonable request.
