# Supplementary material for: Integration of Sustainable Carbon Nanoparticles Into Inverted Hybrid Perovskite Solar Cells to Enhance Optoelectronic Performance
Source: Glob Chall. 2025 Nov 21;10(1):e00363. doi: 10.1002/gch2.202500363 (PMC12774800; doi:10.1002/gch2.202500363)
Supplement: Supplementary file 1 — Supporting file: gch270069‐sup‐0001‐Suppmat.pdf [file GCH2-10-e00363-s001.pdf]

Supporting Information

Integration of Sustainable Carbon Nanoparticles into Inverted Hybrid Perovskite Solar Cells to Enhance Optoelectronic Performance

Lorenzo Squillantini[a,1,], Davide Tocco[b,1], Marco Natali[a*], Luca Gnoli[a], Alessio Mezzi[c], Chiara Dionigi[a], Eugenio Lunedei[a], Fabiola Liscio[a], Andrea Parisini[a], Mirko Seri[a], Samet Ocak[a], Silvia Milita[a], Emiliano Fratini[b], Giampiero Ruani[a], Francesca De Giorgio[a,d,*]*

[a] Lorenzo Squillantini, Marco Natali, Luca Gnoli, Chiara Dionigi, Eugenio Lunedei, Fabiola Liscio, Andrea Parisini, Mirko Seri, Samet Ocak, Silvia Milita, Giampiero Ruani, Francesca De Giorgio
Consiglio Nazionale delle Ricerche, Istituto per lo Studio dei Materiali Nanostrutturati (CNR-ISMN)

Via Piero Gobetti 101, 40129 Bologna, Italy.

E-mail: francesca.degiorgio@cnr.it, lorenzosquillantini@cnr.it, marco.natali@cnr.it

[b] Davide Tocco, Emiliano Fratini
Department of Chemistry ''Ugo Schiff'' & CSGI, University of Florence
Via della Lastruccia 3, 50019, Sesto Fiorentino (FI), Italy

[c] Alessio Mezzi
Consiglio Nazionale delle Ricerche, Istituto per lo Studio dei Materiali Nanostrutturati (CNR-ISMN)
Strada Provinciale 35d, n. 9, 00010 Montelibretti (RM), Italy

[d] Francesca De Giorgio
National Reference Center for Electrochemical Energy Storage (GISEL), INSTM, Via G. Giusti 9, Firenze, Italy, 50121

[1] Lorenzo Squillantini and Davide Tocco contributed equally to this work.

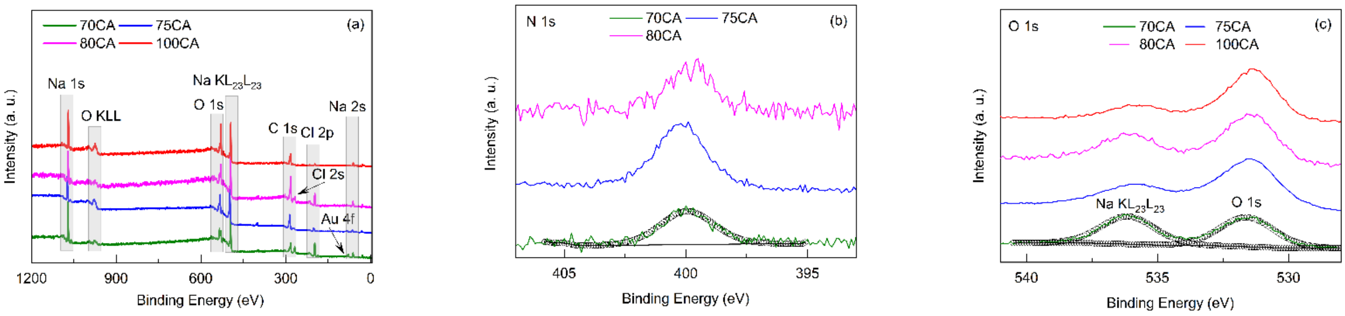

Figure S1. Survey XPS spectra of (a) all the carbon nanoparticles (CNPs) compositions; high resolution spectra of (b) O1s and (c) N1s.

Table S1. XPS quantification (atomic %, at. %), binding energy (B.E.) values and assignment of the chemical states.

CNP		C1s-1	C1s-2	Cl2p	N1s	Na1s	O1s
		C-C	COOH	Cl(-1)	amine	Na(+1)	C-O
100CA	B.E. (eV)	285.0	288.3	198.8	-	1071.8	531.4
	a.t. (%)	32.2	21.2	2.9		12.2	31.5
80CA	B.E. (eV)	285.0	288.2	199.2	399.9	1071.8	531.5
	a.t. (%)	54.1	11.2	12.7	2.6	5.8	13.6
75CA	B.E. (eV)	285.0	288.2	199.3	400.1	1071.7	531.6
	a.t. (%)	41.0	13.5	5.0	4.0	10.4	26.2
70CA	B.E. (eV)	285.0	288.5	199.1	400.0	1071.9	531.6
	a.t. (%)	37.6	9.9	24.2	3.6	10.9	13.8

Table S2. Average J_{SC}, V_{OC}, FF and PCE figure of merit calculated from six different hybrid perovskite solar cells (HPSCs) with 70CA CNPs at different concentrations (0.12 mg/mL, 0.25 mg/mL, 0.50 mg/mL) obtained under solar irradiation in reverse scan. In brackets, the values of each champion device are reported (Fig. 7a).

Additive in HPSC	J_{SC} (mA cm^{-2})	V_{OC} (V)	FF (%)	PCE (%)
No additives	17.7±0.4 (18.7)	0.85±0.01 (0.88)	53.5±1.5 (50)	8.1±0.1 (8.2)
0.12 mg/mL	16.4±0.5 (17.1)	0.80±0.03 (0.85)	52±8 (62)	7.0±1.5 (9.0)
0.25 mg/mL	17.8±0.6 (17.5)	0.86±0.03 (0.90)	60±3.0 (63)	9.2±0.4 (10.0)
0.50 mg/mL	14.8±0.4 (15.3)	0.79±0.01 (0.80)	61±3 (64)	7.1±0.3 (7.8)

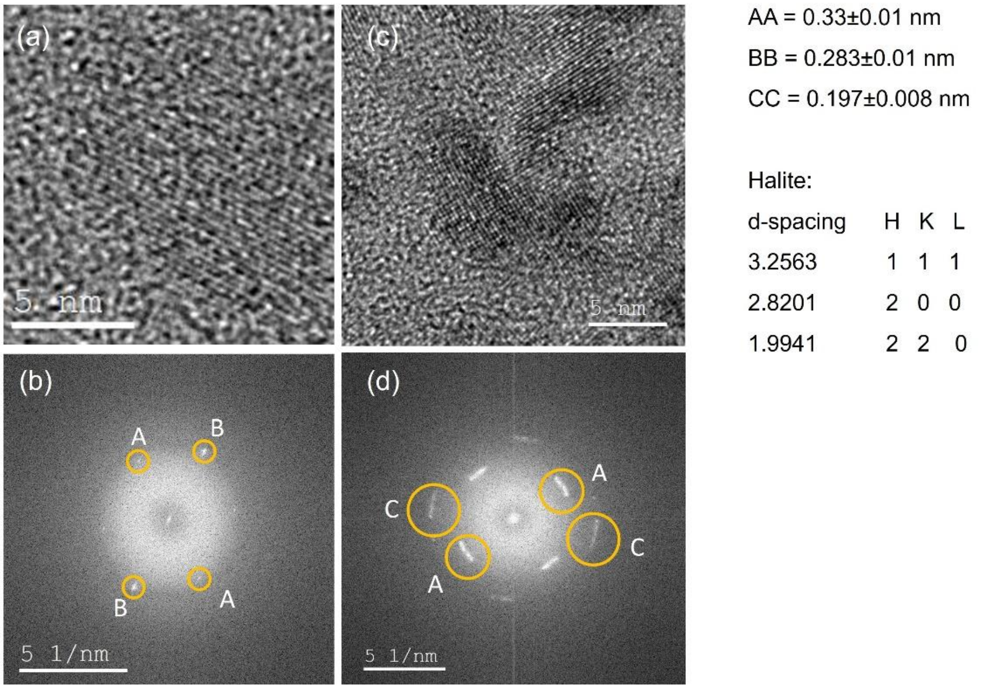

Figure S2. HREM micrographs (a, c) of the 70CA sample and corresponding diffractogram (b, d) showing the presence of Halite NaCl particles. In the rightmost panel, interplanar distances obtained from (b) and (d) are compared with those of the Halite NaCl structure (D. Walker et al., *Am. Miner.* **2004**, 89, 204-210, DOI: 10.2138/am-2004-0124).

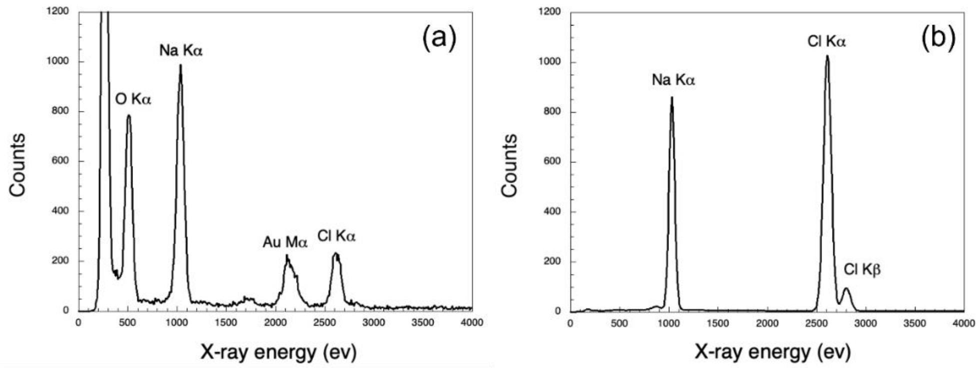

Figure S3. Comparison of the Na Ka and Cl Ka peaks present in (a) an experimental EDS spectrum obtained on the non-dialyzed sample and (b) a simulated EDS spectrum (simulation obtained by using Desk Top Analyzer 2.5.1 N.I.S.T. and N.I.H by C.E. Fiori, C. R. Swit and R. L. Myklebust, 1997) of pure NaCl 20 nm thick. Simulations include all the parameters of our EDS detector. From the simulation in (b), the background subtracted integrated peak Intensity ratio between Na and Cl appears to be less than 1, at variance with what can be obtained experimentally. This indicates the

presence in the non-dialyzed sample of an excess sodium compared to the amount that could be accounted for by the presence of the NaCl particles. In (a), the Au Ma peak arise from the TEM support grid.

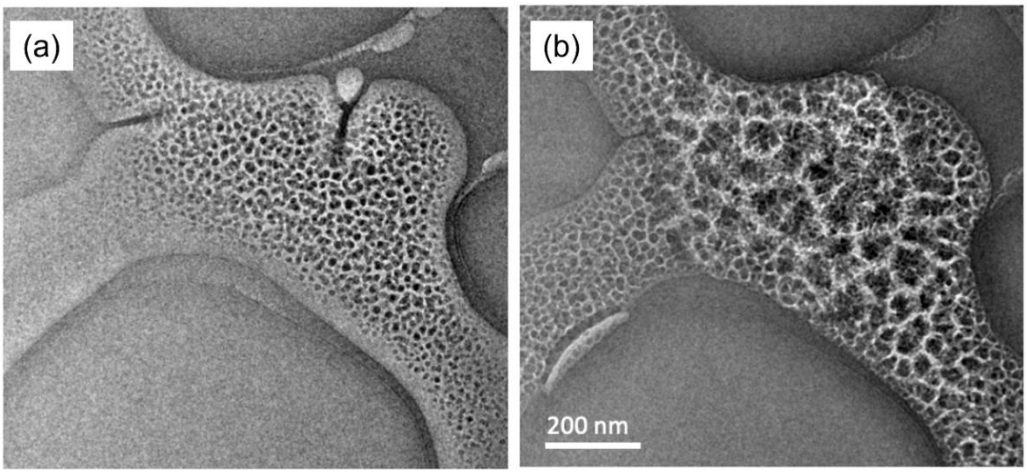

Figure S4. Bright Field (BF) TEM micrographs of the dialyzed 70CA sample before (a) and after (b) an electron irradiation of about 2 min with the beam focused on the central part of the micrographs (beam diameter about 0.5 mm). As an effect of electron irradiation, dark-contrasted particles in (a) are seen to grow in size and coalesce in (b).

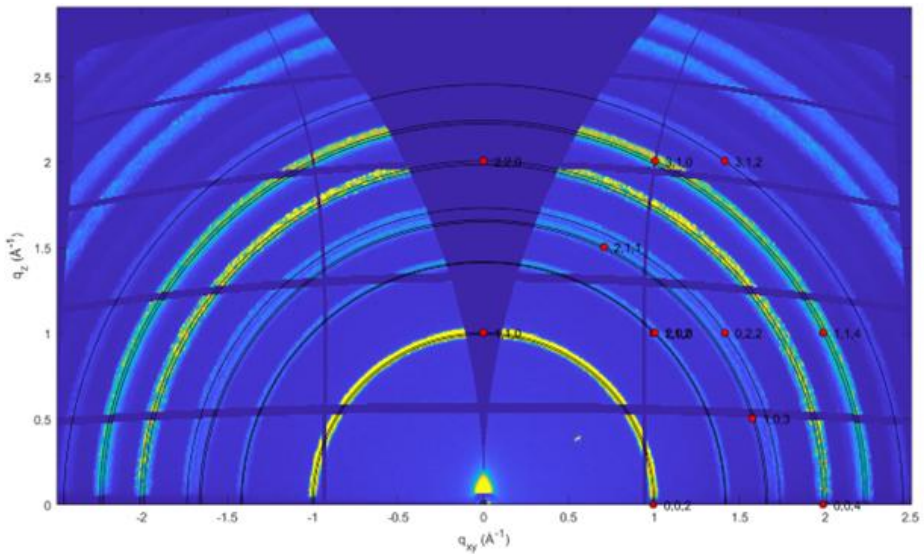

Figure S5. 2D-GIWAXS image of MAPI:CD film on PEDOT:PSS recorded in ambient air by using incident angle equal to 0.3° and X-ray wavelength of 1.4 Å

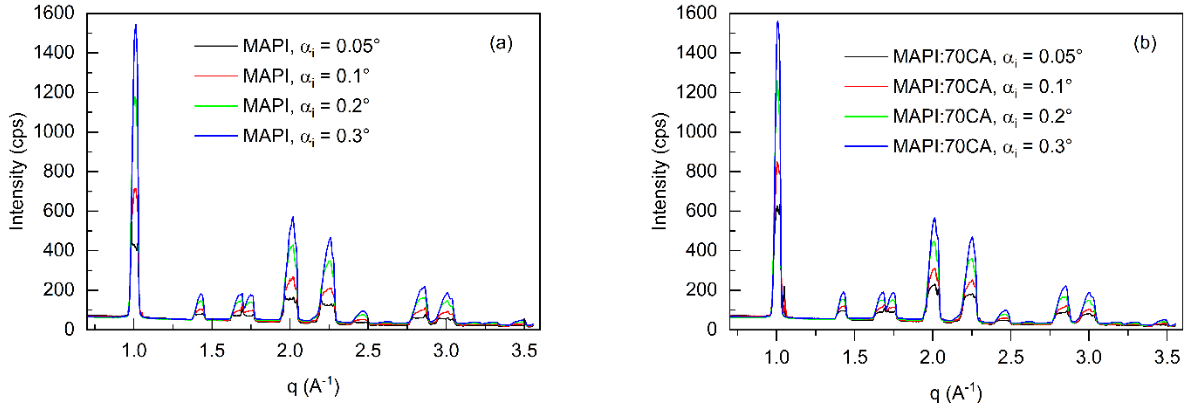

Figure S6. Series of XRD profiles extracted by the 2D-GIWAXS of (a) MAPI and (b) MAPI:70CA films on PEDOT:PSS layers recorded by using different incident angles (from 0.0° to 0.3°) and X-ray wavelength of 1.4 Å in ambient air.

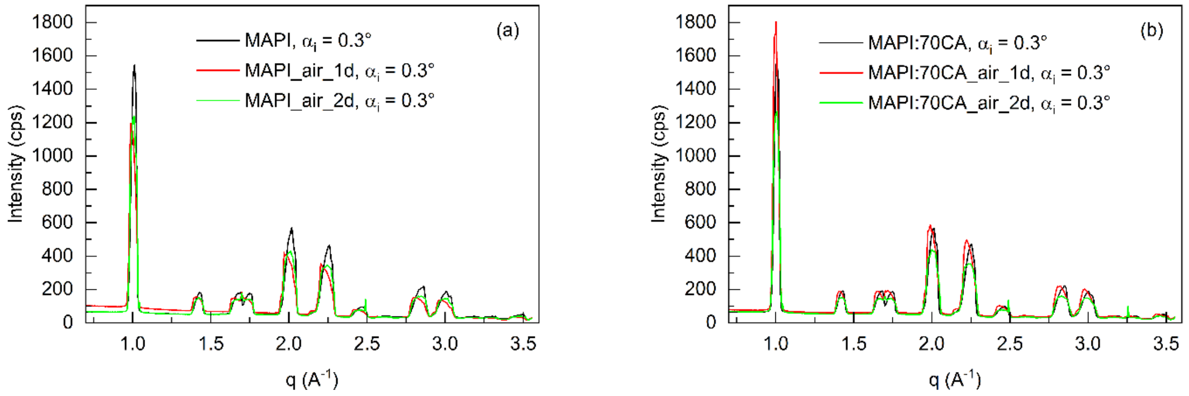

Figure S7. XRD profiles extracted by the 2D-GIWAXS of (a) MAPI and (b) MAPI:70CA films on PEDOT:PSS layers recorded in ambient air after 1 day- (red line) and 2 day-exposure time (green line), by using 0.3° incident angle and X-ray wavelength of 1.4 Å.

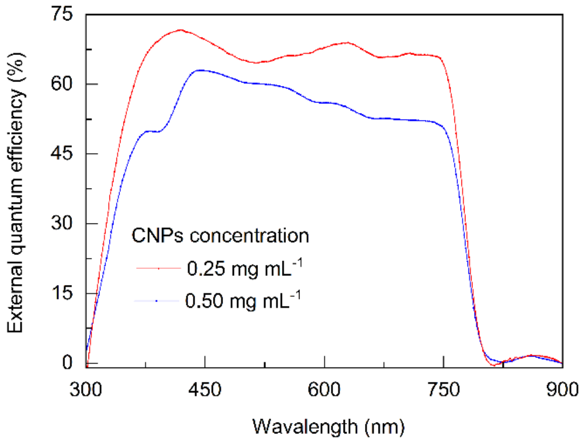

Figure S8. External quantum efficiency (EQE) spectra of HPSCs incorporating CNPs with the optimal 70CA composition at concentrations of 0.25 mg/mL (red line) and 0.5 mg/mL (blue line). The corresponding theoretical short-circuit current densities *(J_{SC})*, calculated by integrating the EQE spectra with the AM1.5G solar spectrum, are 17.1 and 14.3 mA/cm², respectively. These values are in agreement with Table S2.

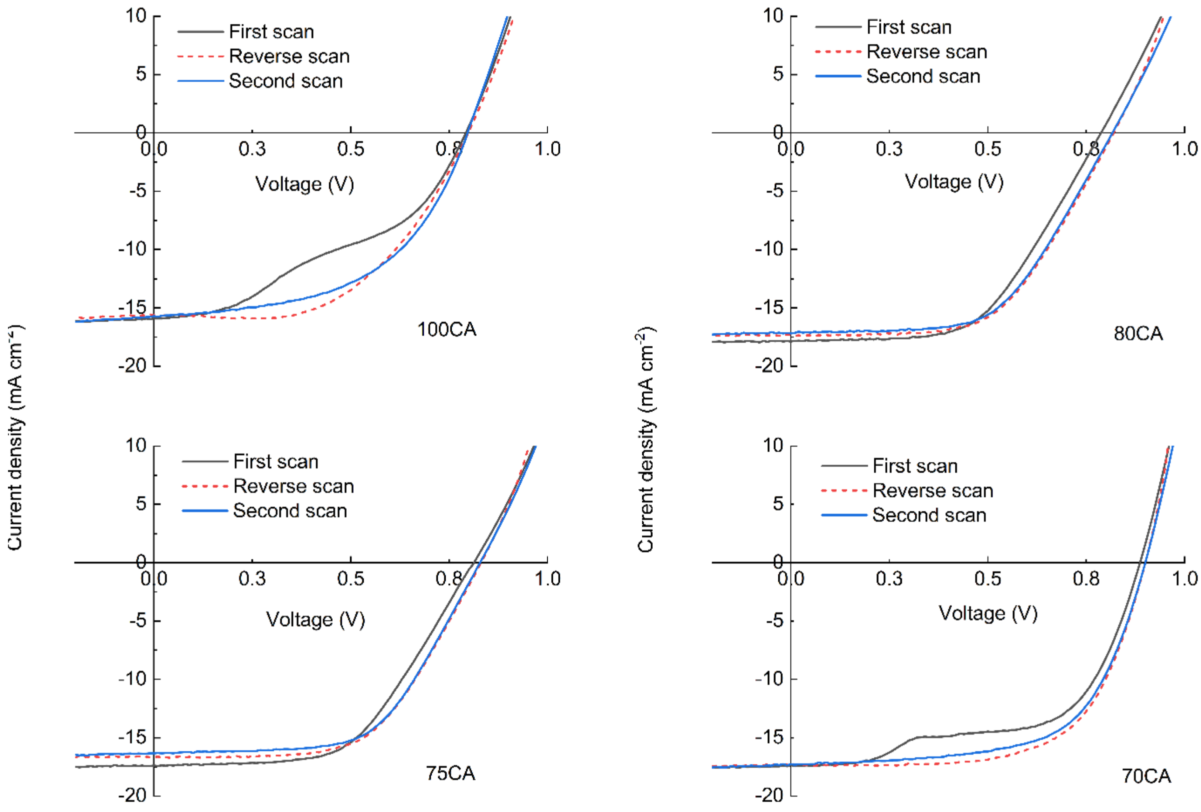

Figure S9. *J/V* curves (forward and reverse scans) obtained under solar irradiation of MAPI-based devices featuring CNPs having different compositions at the fixed concentration of 0.25 mg/mL. Forward scans were performed from -0.5 to 1 V and reverse ones from 1.0 V to -0.5 V at 100 mV/s scan rate.

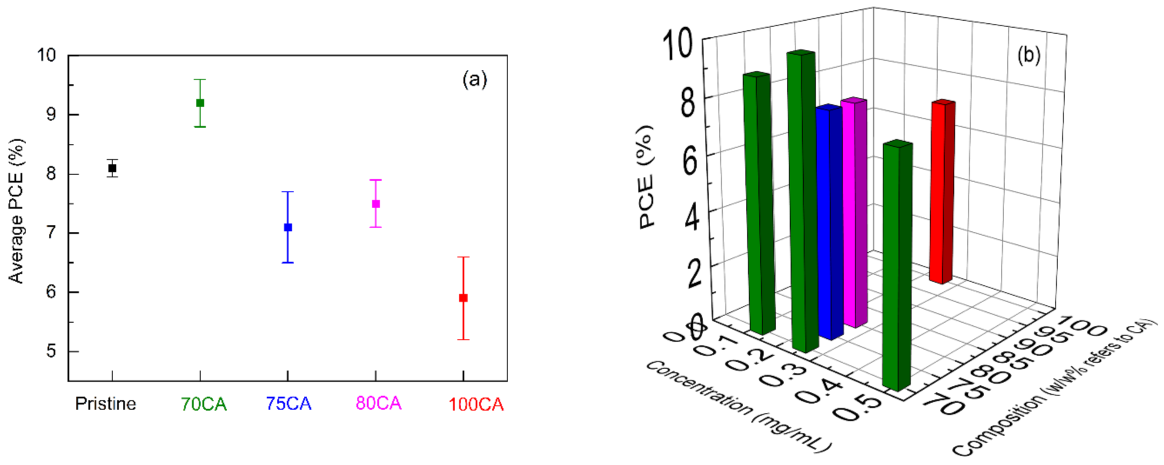

Figure S10. (a) Average PCEs with standard deviations for each CNP composition at 0.25 mg/mL. (b) Histogram bars chart of the champion devices' PCEs for each CNPs composition and concentration.

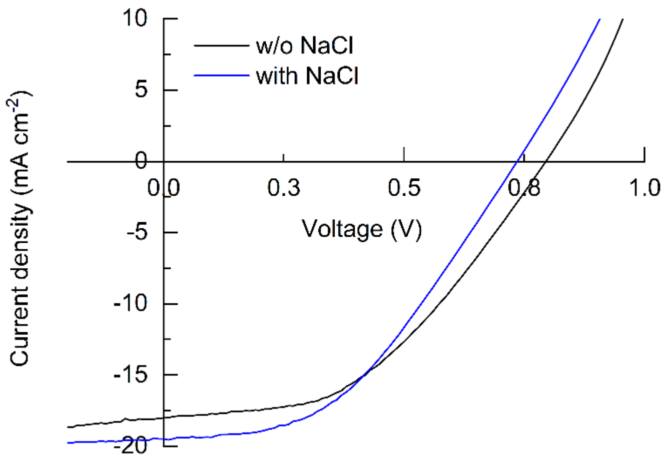

Figure S11. J/V curves under solar irradiation of (a) a pristine MAPI-based device (b) and (b) with NaCl 0.06 mg/mL.

Table S3. Fitting parameters of the impedance spectra of the HPSCs with 0.25 mg/mL CNPs concentration and of the pristine one, collected at different applied voltages (V_{app}).

	V_{app} (V)	R_S (Ω cm^2)	C_D (F cm^{-2})	R_{rec} (Ω cm^2)	R_C (Ω cm^2)	C_1 (F cm^{-2})	R_L (Ω cm^2)	L (H cm^{-2})
70CA	0.9	4.80	$1.32\cdot10^{-7}$	46.4	160.41	$1.08\cdot10^{-7}$	751.41	8830
0.25 mg/mL	0.8	4.49	$8.89\cdot10^{-8}$	123.81	211.21	$1.00\cdot10^{-7}$	423.46	45800
	0.7	4.21	$7.36\cdot10^{-8}$	272.83	278.25	$7.24\cdot10^{-8}$	928.34	116000
Pristine	0.83	5.37	$1.04\cdot10^{-8}$	29.71	121.80	$1.32\cdot10^{-9}$	1159.33	719
	0.7	5.65	$5.80\cdot10^{-9}$	217.34	587	$3.46\cdot10^{-8}$	-	-
